# Antithyroid peroxidase antibodies in women with polycystic ovary syndrome

**Published:** 2013-12

**Authors:** Saghar Salehpour, Nasrin Saharkhiz, Aida Moeini, Anahita Enzevaei

**Affiliations:** *Department of Obstetrics and Gynecology, Infertility and Reproductive Health Research Center, Taleghani Hospital, Shahid Beheshti University of Medical Sciences, Tehran, Iran.*

One of the most common endocrine disorders in women of reproductive age is polycystic ovary syndrome (PCOS) which its prevalence is reported around 6.5-8% (1). Some clinical trials assessed association between PCOS and other autoimmune related endocrinopathies such as impaired glucose tolerance (IGT), type 2 diabetes mellitus and thyroid dysfunction with controversial results (2). Some investigators showed increase level of serum antiovarian antibodies in the half of affected women (3, 4). However, the exact mechanism of autoimmune processes in PCOS pathogenesis is remained to be fully elucidated. Autoimmune thyroid disorder including hashimoto’s thyroiditis also has been shown to have correlation with PCOS (5). According to Janssen’s *et al *study the prevalence of autoimmune thyroiditis (AIT) was three times higher in patients with PCOS compared with control group (6). The cause of this high incidence is open to speculation. Genetic defect was assumed to predispose persons to AIT as well as PCOS. Both disorders seem to have an oligo-genetic background (5, 7). To date, a common genetic background has not been found. Gleicher *et al* hypothesized that development of PCOS could be attributed to functional autoantibodies including thyroid autoantibodies [anti-thyroid peroxidase antibodies (anti-TPO), and thyroglobulin antibodies (TG-Ab)] (8). Ott *et al* reported a poor treatment response in infertile PCOS women with elevated anti-TPO levels (9). 

In a cross-sectional study at Infertility and Reproductive Health Center (IRHRC), we evaluated serum level of thyroid function tests (TFT) including autoantibodies and to assess their relation with other characteristic and hormonal parameters of PCOS. A total of seventy five women with average age of 26 years who fulfilled the 2003 Rotterdam Criteria underwent gynecologic evaluation and blood sampling between January 2009 and December 2011. The mean±SD of serum anti-TPO in Ott *et al*, Ganie *et al*, Kachuei *et al*, Janssen *et al* and our study were 52.2±98.5, 321.4±189.6, 216±428, 123±328, and 41.06±91.18 IU/ml respectively (5, 6, 9, 10). The wide range of reported values could be attributed to different type of laboratory assessment, value of cut point and heterogeneity of selected patients. About fifteen percent of our patients were anti-TPO positive; the reported range of positive anti-TPO percentage was 15 to 30 in different studies (5, 6, 9, 10 and present study). In fact, the observed ratio in our research is lower than Kachuei’s study which held in a same country (5). In that research, authors did not find difference in rate of positivity between PCOS and control group. Percentage of anti-TPO positivity in Ganie *et al* and Janssen *et al* in normal women were zero and eight percent respectively (6, 10). In our study, presence or absence of anti-TPO did not have a major influence on the characteristics and hormonal values of the PCOS patients; only hip circumference and estradiol level were significantly higher in anti-TPO positive cases. In another study this difference was observed for hypoechoic ultrasound pattern, patient’s age and LH to-FSH-ratio. Ott *et al* suggested that anti-TPO could be a good predictive marker for treatment response in infertile women with PCOS (9). They reported a higher anti-TPO level in patients who were resistant to clomiphene citrate treatment. TSH level in our PCOS patients was 3.16±3.55 mU/l; other investigators report its value between 2 and 3 mU/l. We did not find any difference in biochemical and hormonal serum values of patients between euthyroid and hypothyroid ones. TSH level in PCOS patients and control subjects did not differ significantly in Ganie *et al*, Kachuei *et al* researches which is in contrast to the results of Janssen and colleagues (5, 10).

High prevalence of thyroid autoimmunity in PCOS women of reproductive age brings this question in our mind that is it necessary to screen these patients for hypothyroidism? Due to increased risk of morbidity in hypothyroid mother and her newborn, it seems logical to assess thyroid function test in suspicious patients. To have a proper answer for this question, however, several prospective researches should be conducted with more number of participants in a longer follow up period. Based on our data, it is more likely to see a disturbance in serum values of thyroid function test and autoantibodies in women with PCOS. We recommend evaluation of these parameters by longitudinal cohort studies with more number of cases and a longer follow up period.
